# Adenoviral Infections in Neonates: A Case-Based Literature Review

**DOI:** 10.7759/cureus.29082

**Published:** 2022-09-12

**Authors:** Stergiani Keramari, Liana Fidani, Alexandros Poutoglidis, Stefanos Chatzis, Nikolaos Tsetsos, Georgia Kaiafa

**Affiliations:** 1 Department of Paediatrics, University General Hospital of Thessaloniki AHEPA, Thessaloniki, GRC; 2 Department of Medical Biology Genetics, Aristotle University of Thessaloniki, Thessaloniki, GRC; 3 Department of Otorhinolaryngology-Head and Neck Surgery, George Papanikolaou General Hospital, Thessaloniki, GRC; 4 Department of Surgery, 251 Airforce Hospital, Athens, GRC; 5 First Propaedeutic Department of Internal Medicine, University General Hospital of Thessaloniki AHEPA, Thessaloniki, GRC

**Keywords:** clinical symptoms, birthweight, gestational age, neonates, adenoviral infection

## Abstract

Adenoviral infections in neonates are associated with high rates of mortality due to the lack of humoral immunity. A comprehensive search of published literature in PubMed, Google Scholar, and Science Direct electronic databases was conducted for case reports published between the years 1990 and 2021. The aim of our study is to investigate the risk factors, clinical manifestations, treatment, and outcomes of adenoviral infections in neonates. In our study, 36 cases were included. The most common type of infection was disseminated one (14/36, 38.8%), followed by adenoviral pneumonia (13/36, 36.1%). Cidofovir was administered in seven cases (19.4%), and death was reported in six of them. One preterm low birthweight neonate with disseminated adenoviral infection was treated with a combination of cidofovir, intravenous immune globulin, and haploidentical virus-specific T lymphocytes (VSTs) and survived. In this review, we found a statistically significant difference in the outcome based on the type of adenoviral infection (p=0.001). Disseminated infection and pneumonia are associated with the worst prognosis. In addition, mortality was observed to be higher in neonates with disseminated disease in contrast to neonates with localized infection (p=0.002). However, the antiviral treatment had no statistically significant effect on the mortality rate (p=0.137). There is a necessity for further investigation and randomized studies to validate the results of the present study.

## Introduction and background

Introduction

Adenoviral infections have a wide range of manifestations, depending on the patients' immunological status, ranging from asymptomatic to severe infection. The vast majority of cases in immunocompetent hosts are self-limited with rare fatal complications. Adenoviral infection in neonates is not frequent but may be life-threatening. Species A-F are responsible for the outbreaks. The serotypes have a different tissue tropism and thus cause different clinical manifestations. The dominant serotypes differ over time and among regions. Infants and neonates are susceptible to severe disease with fatal complications. Mortality rates may exceed 85% [1-7]. In neonates, the adenoviral transmission may be vertical and horizontal. Ronchi et al. demonstrate that horizontal transmission may be more likely when neonatal symptoms present in the neonatal mean age of 16 ± 8 days after delivery [8-9]. Adenovirus entry takes place by attachment to CAR receptor of trophoblast cells, which varies with gestational age and trophoblast phenotype, leading to transplacental passage of adenovirus, and latent embryo infection. Adenoviruses can cause a wide range of diseases including pneumonia, keratoconjunctivitis, cystitis, nephritis, urethritis, hepatitis, colitis with hematochezia, esophagitis, encephalitis and myocarditis that can progress to disseminated adenoviral infection [10-13]. There is no specific therapeutic antiviral agent.

## Review

Methods 

We performed a comprehensive electronic search of published literature in PubMed, Science Direct and Google scholar databases covering the time span between 3 October 2021 to 20 December 2021. Our search focused on case reports of neonatal adenoviral infections published between 1990-2021. Case reports included the following inclusion criteria: (1) patients' age 0-30 days (2) gestational age and birthweight, (2) onset of neonatal symptoms on the specific day of life, (3) type of infection, (4) adenoviral serotype, (5) treatment and (6) outcomes. Exclusion criteria were: (1) age above 30 days of life, (2) other immunocompromised patients, (3) duplicates, and (4) conference abstracts. All identified studies were screened by two authors. A total number of 36 cases were identified. The is no need for ethics approval because this study is a review of published case reports in the last 30 years (Table 1).

**Table 1:** Characteristics of reported cases in neonates with adenoviral infection M: male, F: female, ADV: adenoviral, GA: gestational age, IVIG: intravenous immunoglobulin, VST: virus-specific T lymphocytes, N/A: not available

ARTICLE	YEAR	GENDER	GA	WEIGHT (gr)	ADV INFECTION	ONSET OF SYMPTOMS (DAY)	ADV SEROTYPE	ANTIVIRAL TREATMENT	OUTCOME
Matsuoka et al. [27]	1990	M	40w	3410	Disseminated	Day 3 of life	19	NO	Death
Brown et al. [23]	1991	M	>38w	N.A.	Disseminated	Day 4 of life	35	N/A	Death
Brown et al. [23]	1991	M	32w	1190	Pneumonia	Day 1 of life	35	N/A	Death
Laungani et al. [37]	1991	N/A	37w	4000	Pneumonia	Day 3 of life	N/A	NO	Alive
Laungani et al. [37]	1991	N/A	37w	1320	Pneumonia	Day 4 of life	N/A	NO	Death
Osamura et al. [31]	1992	N/A	38w	2768	Disseminated	Day 6 of life	11	NO	Death
Pinto et al. [36]	1992	N/A	39w	3680	Pneumonia	Day 7 of life	35	NO	Death
Kinney et al. [39]	1994	M	41w	3770	Pneumonia	Day 0 of life	7	NO	Alive
Kinney et al. [38]	1994	M	40w	4020	Cystitis	Day 0 of life	N/A	NO	Alive
Chiou et al. [24]	1994	M	40w	1805	Disseminated	Day 1 of life	6	NO	Death
Montone et al. [29]	1995	N/A	25w	660	Pneumonia	Day 1 of life	N/A	NO	Death
Debast et al. [36]	1996	N/A	38w	3740	Pneumonia	Day 5 of life	N/A	IVIG	Alive
Kim et al. [40]	1997	M	36w	2700	Pneumonia	Day 3 of life	N/A	NO	Death
Kim et al. [40]	1997	F	35w	1830	Pneumonia	Day 0 of life	N/A	NO	Death
Kim et al. [40]	1997	F	43w	5100	Pneumonia	Day 0 of life	N/A	NO	Death
Aebi et al. [41]	1997	M	38w	3300	Disseminated	Day 10 of life	N/A	Ribavirin	Death
Rieger-Fackeldey et al. [32]	2000	M	35w	2540	Disseminated	Day 6 of life	N/A	N/A	Death
Rieger-Fackeldey et al. [32]	2000	F	36w	2760	Conjunctivitis	Day 4 of life	N/A	IVIG	Alive
Gavin et al. [26]	2002	F	N/A	N/A	Pneumonia	Day 14 of life	2	Ribavirin	Alive
Gavin et al. [26]	2002	F	N/A	N/A	Pneumonia	Day 8 of life	N/A	Ribavirin	Death
Elnifro et al. [20]	2005	F	N/A	N/A	Conjunctivitis	Day 18 of life	8	NO	Alive
Elnifro et al. [20]	2005	M	35w	1900	Conjunctivitis	Day 8 of life	8	NO	Alive
Elnifro et al. [20]	2005	F	38w	3000	Conjunctivitis	Day 5 of life	40	NO	Alive
Cichocki et al. [42]	2007	N/A	40w	3530	Colitis	Day 1 of life	N/A	NO	Alive
Henquell et al. [2]	2009	F	>38w	3360	Disseminated	Day 10 of life	15,9	N/A	Death
Kelley et al. [43]	2010	N/A	32w	1870	Disseminated	Day 8 of life	N/A	Cidofovir+IVIG	Death
Fukuda et al. [25]	2011	F	40w	2242	Colitis	Day14 of life	31	NO	Alive
Soileau et al. [34]	2013	F	40w	N/A	Disseminated	Day 4 of life	11,7	Cidofovir	Death
Baserga et al. [22]	2018	F	39w	3500	Colitis	Day 3 of life	40,41	NO	Alive
Censoplano et al. [33]	2018	F	39w	N/A	Pneumonia	Day 10 of life	N/A	Cidofovir	Death
Censoplano et al. [33]	2018	F	40w	N/A	Disseminated	Day 9 of life	N/A	Cidofovir+IVIG	Death
Tamiya et al. [19]	2019	M	38w	2.635	CNS infection	Day 24 of life	40,41	NO	Alive
Auletta et al. [30]	2019	F	29w	1480	Disseminated	Day 1 of life	C	Cidofovir+IVIG+Haploidentical VSTs	Alive
Otto et al. [35]	2021	F	23w	N/A	Disseminated	Day 8 of life	15,29	Cidofovir+IVIG	Death
Otto et al. [35]	2021	M	>38w	N/A	Disseminated	Day 9 of life	56	Cidofovir	Death
Otto et al. [35]	2021	M	>38w	N/A	Disseminated	Day 14 of life	56	IVIG	Death

Neonates were classified into three groups, based on (1) gestational age (term neonates ≥37 weeks gestational age and preterm neonates <37 weeks gestational age), birthweight (low birthweight <2500 g, normal birthweight: 2500-4000 g, high birthweight >4000 g) and the age at onset of symptoms (at the age <8 days of life at the onset of symptoms and at the age ≥8 days at the onset of symptoms). 

The data were collected on an Excel sheet (Microsoft Corporation, Redmond, WA). Statistical analysis was performed using Fisher’s exact test and a chi-square test. We compared gestational age, birthweight, the onset of symptoms, type of adenoviral infection and treatment between survivals and deaths. Student’s t-test was used for continuous variables like the mean age of neonates. Continuous data approximating the normal are presented with standard deviation. The graphs were created using GraphPad Prism software version 9.3.0 (GraphPad Software, La Jolla, CA). A p-value <0.05 was considered to be significant. 

Results 

In our review, 36 published cases of adenoviral infection in neonates were included. The median age was of 6.3 days +/- 5.4, 15 were female (41.6%) and 23 of them were term neonates (63.8%). The most common adenoviral infection was disseminated, (14/36, 38.8%), followed by adenoviral pneumonia (13/36, 36.1%), adenoviral conjunctivitis (4/36, 11.1%), adenoviral colitis (3/36, 8.3%), central nervous system adenoviral infection (1/36, 2.7%), and adenoviral cystitis (1/36, 2.7%). 

**Figure 1:**
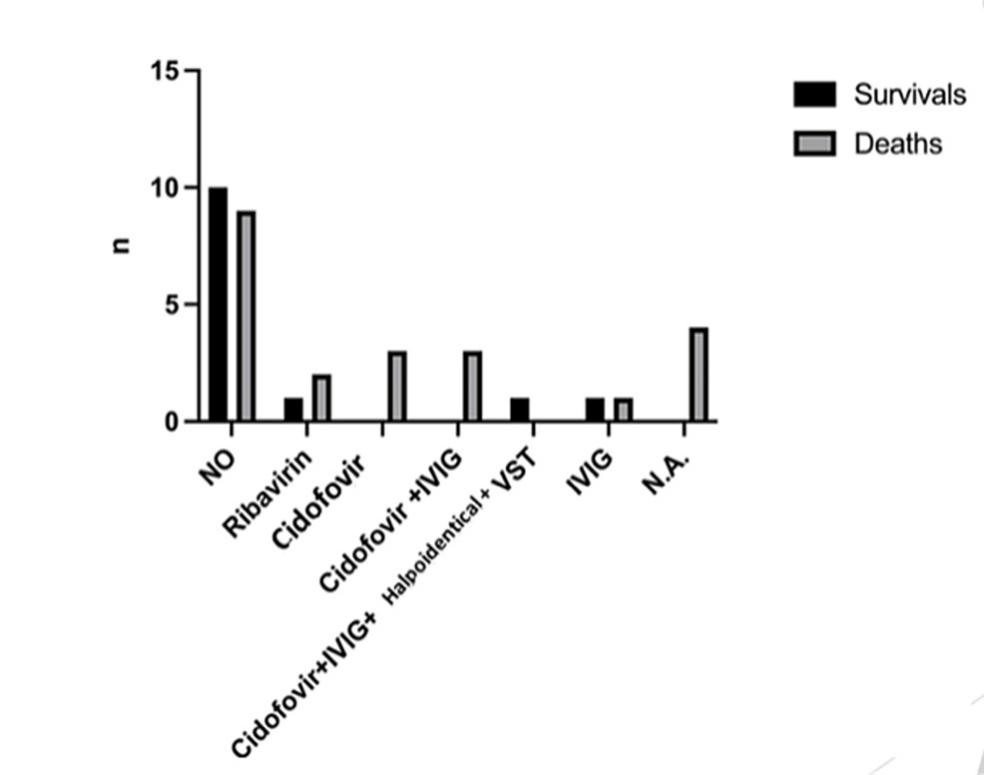
Outcomes in relation to the antiviral treatment used IVIG: intravenous immunoglobulin, VST: virus-specific T lymphocytes, NA: not available

The most common antiviral drug used was cidofovir in seven cases (19.4%), either as monotherapy (3/36) or combined with intravenous immunoglobulin (IVIG) (3/36) and death was reported in all of them. One preterm low birthweight neonate having disseminated infection was treated with a combination of cidofovir, IVIG, and haploidentical virus-specific T lymphocytes (VSTs) and survived. In particular, there was a 100% mortality rate in the monotherapy group and an 83.3% mortality rate in the combined treatment with the IVIG group. Other treatment options included ribavirin (3/36, 8.3%) and IVIG administered intravenously (2/36, 8.3%). The mortality rate in the neonates treated with ribavirin was 100% (two of them had an adenoviral lung infection and one of them had disseminated infection) and in those treated with IVIG, the mortality rate was 50%. There was a percentage as high as 52.7% of neonates who received no antiviral treatment; the outcome was an improvement in 27.7% of them. The therapies of four patients were not mentioned. In addition, in cases, where the therapeutic strategy was unknown, death was recorded in all of them. There was no statistically significant difference in the outcome compared to the antiviral treatment used (x2=9.71, df=6, p=0.137) (Figure 1).

However, we found a statistically significant difference in the outcome of disseminated infection compared to the antiviral treatment used (x2=14.00, df=5, p=0.015).

**Figure 2:**
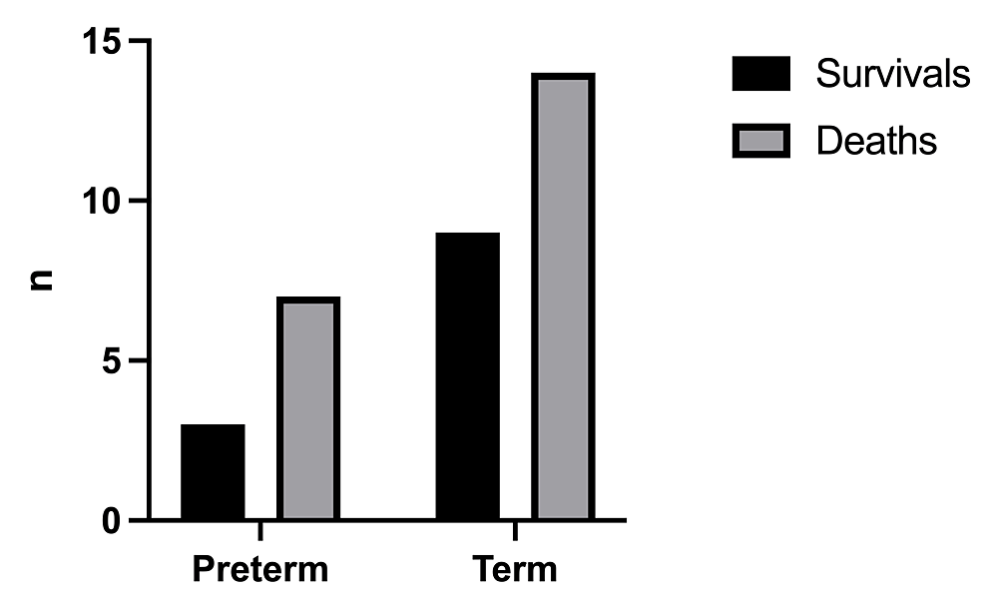
Outcomes in relation to gestational age Term neonates ≥37 weeks gestational age, preterm neonates <37weeks gestational age

**Figure 3:**
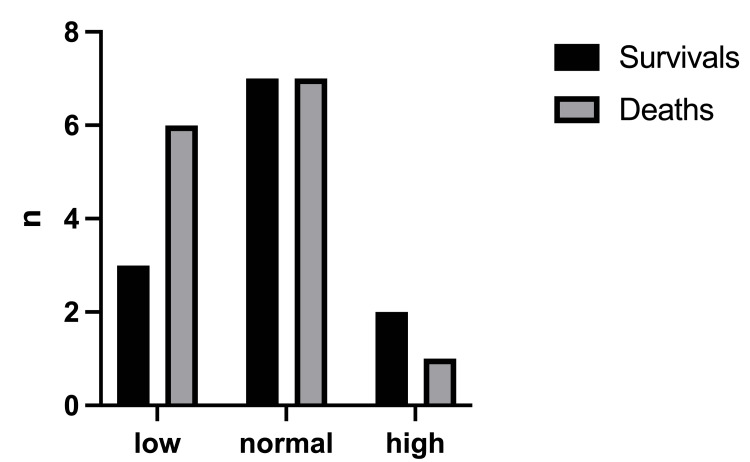
Outcomes in relation to the neonates' birthweight Low birthweight <2500g, normal birthweight: 2500-3999g, high birthweight>= 4000g

**Figure 4:**
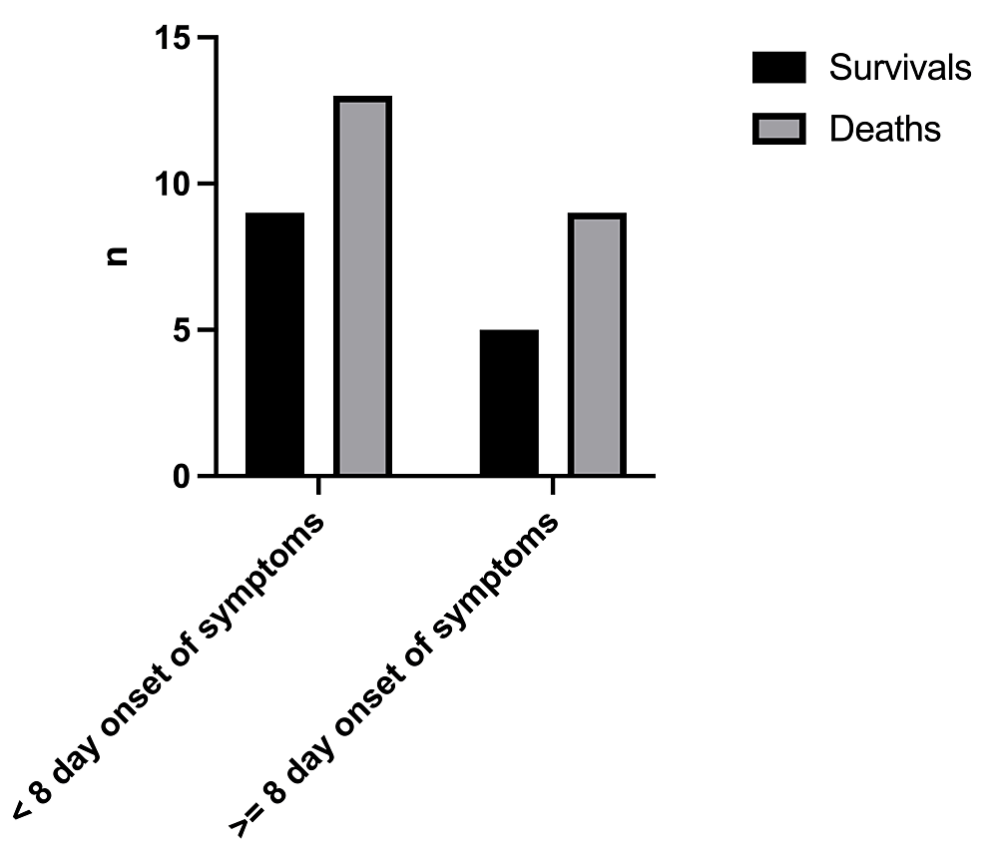
Outcomes in relation to age at onset of symptoms Age <8 days of life at the onset of symptoms, Age ≥8 days at the onset of symptoms

The mortality rate was 61.1% with a total number of deaths of 22/36 (Figure 2). The birthweight (Figure 3) had a significant effect on the mortality rate (x2=0.25, df=1, p=0.502 and x2=1.19, df=2, p=0.552, respectively). In addition, there was no statistically significant difference in the outcome with respect to the age of onset of symptoms (x2=0.098, df=1, p=0.755) (Figure 4).

**Figure 5:**
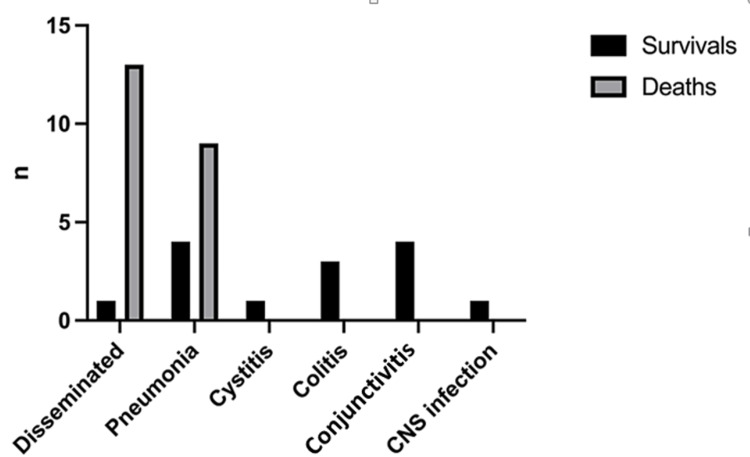
Outcomes in relation to clinical manifestation of the adenoviral infection CNS: central nervous system

Regarding the type of infection, the highest mortality rate was in neonates with disseminated infection (13/14, 92.8%), followed by patients with adenoviral pneumonia (9/13, 69.2%). All other neonates with adenoviral infection survived. There was a statistically significant difference in the outcome based on the manifestation of infection (x2=20.44, df=5, p=0.001) (Figure 5). However, it seems that disseminated adenoviral infection does not depend on gestational age, birthweight, and age of onset of the symptoms (p=1.00, 0.672, 0.314 respectively).

Discussion 

The majority of adenoviral infections in infants and children occur between the ages of six months to four years, and seropositivity may be as high as 80%, due to incomplete humoral immunity [1-3]. Human adenoviruses are nonenveloped, double-stranded DNA viruses belonging to the Adenoviridae family [1-3]. Adenoviral receptors are the following: coxsackievirus B and adenovirus receptors (CAR), membrane cofactors CD46 and the cell adhesion proteins desmoglein 2 (DSG2) as attachment receptors [4-5]. Adenoviruses have been classified into 7 species (A to G) and 55 serotypes. In the immunocompetent host, infections are typically caused by exposure to infected individuals via aerosolized droplets, direct conjunctival secretions, and by the faecal-oral route, because they are resistant to gastric fluids and spread with water, airflow filters, or environmental surfaces. The adenoviral incubation period depends on the serotype and ranges from two days to two weeks [6-8]. Adenoviral infection in neonates is not frequent but may be life-threatening. In neonates, the adenoviral transmission may be vertical and horizontal (from the mother or another family member). Horizontal transmission may be more likely when neonatal symptoms are presenting in the neonatal mean age of 16 ± 8 days. If maternal symptoms started prior to delivery or a few days after delivery and the presence of neonatal symptoms within the first days of life suggests possible ventricle transmission [8-9]. Adenoviruses are very stable viruses and are easily disseminated to neonates from respiratory tract secretions, contaminated hands, surfaces, and equipment.

Vertical transmission has been demonstrated by PCR in the amniotic fluid of a complicated pregnancy, like hydrops and embryo tachyarrhythmia [10]. Adenoviruses were detected in the amniotic fluid of 2% of normal pregnancies [11-12]. Our knowledge is poor about the molecular mechanisms of placental viral infections. The placenta plays a significant role in the vertical transmission of viruses. Adenovirus entry is taken place by attachment to the CAR receptors of extravillous trophoblast cells. The expression of CAR on trophoblast cells differs according to the gestational age and the trophoblast phenotype. Consequently, we concluded that adenoviral infection of the placenta leads the mother and fetus to adverse outcomes and latent infection of the fetus [13].

Clinical manifestations depend on the immunologic status; it can be from asymptomatic infection to fatal infection. In healthy children, adenoviral infections are usually self-limited. Clinical manifestations of adenoviral infections in healthy individuals include non-specific flu-like symptoms [14]. In rare conditions, adenoviral infections in the respiratory tract can cause hemorrhagic pneumonia, respiratory distress syndrome, and severe respiratory failure. Severe respiratory failure accounts for 10-30% of cases [14].

Adenoviruses cause complications either in the gastrointestinal tract such as hemorrhagic colitis combined with hematochezia hepatitis, pancreatitis or in the urinary tract such as hematuria leading to hemorrhagic cystitis. Adenoviral urinary tract complications can lead to renal failure and necrotizing tubulointerstitial nephritis. Disseminated adenoviral infections are associated with high mortality rates, mainly in bone marrow transplanted recipients and neonates [15]. Detection of adenovirus with direct or indirect immunofluorescence assays, viral cultures or PCR of the infected sites (swabs, bronchopulmonary fluids, blood, urine, stool) establish the diagnosis of adenoviral infection. PCR is a useful way to monitor the response to therapy by quantification viral load [13, 16]. Diagnosis of disseminated adenoviral infection may be confirmed by detection of adenoviruses in two or more sites or when adenovirus is isolated from the blood by PCR [16-17]. No specific therapy for adenoviral infection has been approved. Reduction of the viral load has been observed in patients treated with cidofovir, ribavirin, or IVIG. The most preferred therapeutic antiviral agent is cidofovir due to its effective in-vitro activity against adenoviruses. Recently, virus-specific T-cell lymphocytes have been used to treat infections in bone marrow transplants, mainly in pediatric settings, and particularly, in infants with immunodeficiencies. Virus-specific lymphocytes seem to be effective against viral infections but T-cell specific to adenovirus are difficult to be produced due to a large number of adenoviral serotypes. There are reports describing the transfer of maternal adenovirus-specific T-cell lymphocytes to treat disseminated adenoviral infection in neonates [15,18].

In our study, we divide our population into three groups, based on gestational age, birthweight, and age at onset of symptoms. Our aim is to investigate risk factors, clinical manifestations, treatment, and outcome of adenoviral infection in all these previous subgroups of neonates from the published case reports between 1990 and 2021 [19-43]. This is a review of 36 reported cases of adenoviral infections in neonates. The present review indicated that the prognosis of neonatal adenoviral infections correlates with the type of infection. The overall mortality was 61.1% (22/36), but 92.8% (13/14) was for disseminated disease versus 50.0% (9/22) for localized infection (p=0.002). There was no statistically significant difference in the disseminated infection with respect to gestational age, birthweight, and the age at onset of the symptoms. However, it seems there is a statistically significant difference in the outcome compared to the antiviral treatment for disseminated disease, but not for all clinical manifestations of adenoviral infections [44]. 

There are limitations in our present study. The results come only from a limited number of case reports in the literature. Furthermore, in some neonates, gestational age, birthweight and treatment were not available. A small patient group may not have been powered to determine the statistical significance difference. Another limitation is that case reports in literature may present cases with unusual characteristics and are more likely to be unique conditions. All these could alter the results. 

## Conclusions

Adenoviruses are very common and widespread viruses. In neonates, they are associated with high morbidity and mortality rates. Our present study is a review of all the neonatal cases of adenoviral infections published in the last 30 years. This is the first study analyzing these cases in groups, in order to draw conclusions.

The results of our study showed that adenoviral infections represent a life-threatening condition in neonates with high mortality rates. There is no approved antiviral therapy for adenoviral infection. No statistically significant differences were found with respect to the antiviral treatment used. It should be emphasized that there were improvements in premature low birthweight neonates with disseminated infection treated with a combination of cidofovir, IVIG and haploidentical VSTs (except in one case). Taking into account the severity of those infections in neonates, there is a need for further investigation into new antiviral therapeutic strategies such as promising treatment with haploidentical VSTs and a better understanding of the disease.
